# Influence of lowland forests on subsurface salt accumulation in shallow groundwater areas

**DOI:** 10.1093/aobpla/plu054

**Published:** 2013-12-02

**Authors:** Tibor Tóth, Kitti Balog, András Szabó, László Pásztor, Esteban G. Jobbágy, Marcelo D. Nosetto, Zoltán Gribovszki

**Affiliations:** 1 Institute for Soil Sciences and Agricultural Chemistry, Centre for Agricultural Research, HAS, Herman O u. 15.,1022 Budapest, Hungary; 2 Grupo de Estudios Ambientales, IMASL, Universidad Nacional de San Luis and CONICET, Avenida Ejercito de los Andes 950 (5700), San Luis, Argentina; 3 Institute of Geomatics and Civil Engineering, University of West Hungary, 9400 Sopron, Bajcsy-Zsilinszky u. 4., Hungary

**Keywords:** Biomass, salt accumulation, water table level, water uptake.

## Abstract

In flat sedimentary plains in areas with a sub-humid climate, tree planting on grasslands and arable lands creates strong hydrological shifts. As a result of deep rooting and high water uptake of trees, groundwater levels drop and subsurface salt accumulation increases. Tree planting has expanded globally and in Hungary it reached rates of 15 000 ha year^−1^, being focused mainly in the Great Hungarian Plain where forests replace grasslands and crops in a region with widespread shallow groundwater. We performed soil and groundwater observations in 31 pairs of forest and control plots in the region, including gradients of initial water table depth and salinity, soil layering, and tree species and age. Accumulated tree biomass was positively correlated with soil salinization rates following tree planting, being also affected by species (poplar > common oak > black locust) and stand age. Differences among tree species effects appeared to be related to their growth rates. Due to downward deep percolation and salt leaching episodes during the Hungarian winters, the observed salt accumulation rates were lower than those described under similar settings in the warmer Argentine Pampas.

## Abstract (precis)

In flat sedimentary plains in areas with a sub-humid climate, as a result of deep rooting and high water uptake of trees, groundwater levels drop and subsurface salt accumulation increases under tree plantations. Tree planting has expanded globally and its effects were studied in the Great Hungarian Plain where forests are planted in a region with widespread shallow groundwater. Accumulated tree biomass was positively correlated with soil salinization rates following tree planting, being also affected by species (poplar > common oak > black locust) and stand age. Differences among tree species effects appeared to be related to their growth rates.

## Introduction

**Figure 1.**
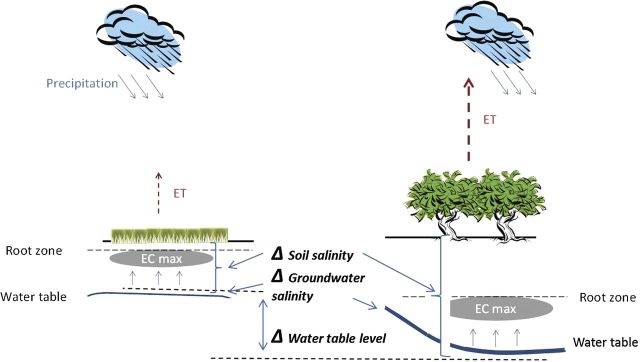
Impact of forest vegetation on water and salt balance of a shallow groundwater site (hypothetical model) after Szabó *et al.* (2012). Also indicated are the most important variables interpreted in the paper, as the difference between soil and groundwater salinity and water table level between the forest and control stands. ET is evapotranspiration and EC is the electrical conductivity, which was used as a proxy of salinity.

Replacing arable lands and grasslands with planted forests has large effects on ecosystem-level processes, such as increasing primary productivity, acidifying soils, regulating fire dynamics, influencing invasive species occurrence and affecting stream flows, among others (Jobbágy *et al.* 2006). Forest evapotranspiration (ET) is generally higher than the ET of neighbouring grasslands, because of increased leaf area and canopy roughness and deeper root systems of woody vs. herbaceous vegetation (Calder 1998; Nosetto *et al.* 2005). Where shallow water tables are present, trees often use groundwater to complement their water needs. Under the sub-humid climate of the Great Hungarian Plain, where precipitation is generally not enough to support woody vegetation, trees can survive long rainless periods only if they access and consume groundwater (Ijjász 1939; Magyar 1961). In a humid climate, the overall hydrological effect of planted forests does not typically cause concern (Nosetto and Jobbágy 2014), but in sub-humid areas (i.e. aridity index between 0.5 and 0.65, UNEP 1992) the hydrological impacts of replacing open arable lands/grasslands by planted forests raise critical issues, particularly where water tables are close to the surface and may become a significant water source for plant transpiration (up to 300 mm year^−1^ depending on the hydraulic properties of the aquifer as reported by Jobbágy and Jackson 2004). Under these conditions, planted forests can modify the water and salt balance of the grasslands/croplands that they replace (Nosetto *et al.* 2007), causing drops in the water table levels (Major 2002) and increasing salt concentration in soils and groundwater (Nosetto *et al.* 2008) (Fig. 1). Although plantations can achieve higher production rates in areas with access to groundwater, they often introduce the cost of salinization of soil and water resources (Jobbágy and Jackson 2004). The guiding hypothesis presented in Fig. 1 assumes that planting forests will result in a fall of water table level, increased salt and groundwater salinity compared with grasslands and arable lands. Consequently, the effect of planting forests is clearly shown by the values of the three derived variables, which are the differences between water table depth, soil salinity and groundwater salinity of the forested and control stands.

At present, tree planting is rising worldwide and there is a strong need to assess its consequences. The European Union (EEC 1992) is now subsidizing tree planting (EC Regulation 2080/92) and as a result large-scale afforestation is taking place in several countries. The forest cover in Hungary has almost doubled during the last 100 years (1.1–2 Mha) (Standovár 2012, p. 25) and the European Union is favouring an even faster growth of the tree planted areas at rates of 15 000 ha year^−1^ (EC 1999; Andrasevits *et al.* 2005). The areas available for planting trees have generally relatively low economic profitability for annual crop production. Based on an analysis of soil types of formerly forested areas, Führer and Járó (2005) stated that the Great Hungarian Plain can be one of the most important areas for planting forests in the country because of the soil protection and landscape improvement purposes of forests. More than 75 years ago, Ijjász (1939) reported that in the Great Hungarian Plain the groundwater levels are deeper during the entire year under forests that reach the water table compared with grasslands, where the differences increase during the growing season. Jobbágy and Jackson (2007) stated that in the Argentine Pampas these groundwater level differences can be as large as 75 cm. Major (2002) observed that under a 2-km wide coniferous forest plot in Kiskunság area in Hungary the water table level was 0.8–1.1 m deeper than under the neighbouring non-forested areas. There is growing concern that industrial poplar plantations in western Hungary, which can sink water table levels as much as 2–3 m, are limiting crop growth in nearby plots by 40–50 % (Markó 2014).

**Figure 2.**
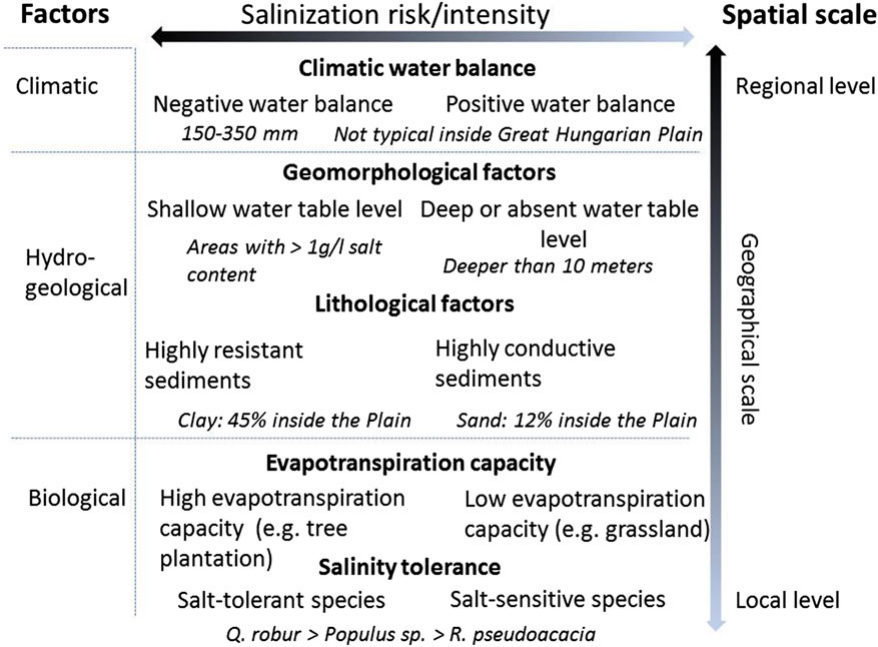
Framework for predicting salinization following vegetation changes, updated from Nosetto *et al.* (2008). The climatic water balance defines the possibility of salinization at the regional scale. But hydrogeological factors (geomorphology and lithology) affect salinization from landscape to regional scales, restricting salinization to areas where groundwater can be accessed and used at significant rates by plants. Biological factors dictate the intensity of salinization across the landscape through the regulation of ET rates and salinity tolerance thresholds.

Different factors affecting salt accumulation in forested areas established over shallow water tables were incorporated into a conceptual model (Nosetto *et al.* 2008) and evaluated in the Argentine Pampas and East Hungary (Nosetto *et al.* 2007). Briefly, this model states that the climatic water balance (precipitation—potential ET) is a first-order factor, influencing salinization at the regional scale. The more negative the water balance the stronger the salinization process. Secondly, lithology and geomorphology act as filters on climate, restricting the extent of salinization to areas where groundwater can be accessed and used at significant rates by plants. Finally, biological factors dictate the intensity of salinization and its location across the landscape, as well, by influencing maximum ET and salinity tolerance of the tree species. The general hypotheses were confirmed by the observations in the Pampas and here we adapted this scheme for the conditions of the Great Hungarian Plain (Fig. 2). We further used them as a guide for the selection of grasslands/trees vs. plantation paired sites and the design and interpretation of multiple soil and groundwater observations.

In this paper, we assess potential salinization threats arising from tree planting on grassland and cropland areas in the Great Hungarian Plain. We compared our results with those found for the same vegetation replacement in the Argentine Pampas, where the existing conceptual model that guided our work was developed. Both the Great Hungarian Plain and the Pampas have a very flat sedimentary terrain, sub-humid climates and widespread shallow water tables. The most important differences between the Great Hungarian Plain and the Pampas include (i) lower mean annual temperature and precipitation (11 vs. 15 °C and 560 vs. 870 mm year^−1^, respectively), which compensate each other resulting in similar climatic water balances; (ii) deeper water tables as a result of intensive drainage interventions more than a century ago (4.5 vs. 1.5 m of depth); (iii) higher soil texture variations and (iv) choice of deciduous tree species (Table 1) given the cold winter constraints as opposed to predominant evergreen species. Given these differences and the conceptual model described above, we anticipated lower salinization risks in the Great Hungarian Plain than on the Argentine Pampas. We expected that colder winters in Hungary and the deciduous tree species would favour winter recharge/leaching event. Moreover, deep water tables would diminish groundwater consumption, thereby also decreasing salt accumulation from groundwater. We also expected important effects of vegetation species given their contrasting growth and water consumption rates. Compared with the studies in the Pampas we investigated how the growth-related parameters of stands, such as total biomass, water uptake and vigour of growth, would affect the accumulation of salts.

**Table 1.** The most important characteristics of the studied forestry species (based on Gőbölös 2002, Magyar 1961 and Szodfridt 1993).

Factors	Species		
	Common oak	Black locust	Poplar
Scientific name	*Quercus robur* L.	*Robinia pseudoacacia* L.	*Populus* × *canadensis* Moench
Climate	No restrictions	Not cold	Not cool
Water demand	High	Unsaturated soil	High
Preferred water table depth (cm)	80–200	Not required	80–200
Soil texture	No restrictions	Sandy	Sandy
Salt tolerance (dS m^−1^)	19	8–10	10
Reported water uptake (mm/vegetation period)	441	273	680
Rotation length (year)	120	60	30
Growth rate	Slow	Fast	Very fast

## Methods

**Figure 3.**
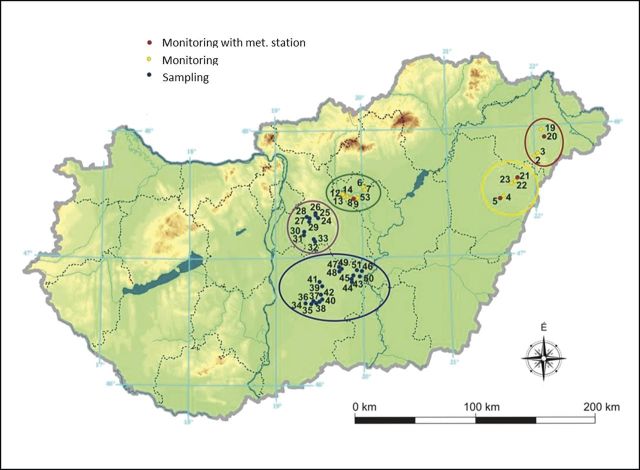
Location of the sampling sites in the Great Hungarian Plain. Five sampling regions inside Hungary were considered: Nyírség, Hajdúság, Jászság, Central Danube Region and Kiskunság. At each sampling site, boreholes of one control plot and associated forest(s) were established. The yellow markers show the monitoring wells, the red markers show monitoring wells + meteorological stations and the blue markers show sampling but not monitored boreholes. Map sections were made by Google Earth application.

The sampling sites (Fig. 3, Tóth *et al.* 2001) were selected on the basis of categories of geologic map units of surface sediments and soils (Kuti *et al.* 1981, 1982, 1984, 1986) based on parameters known to influence the accumulation of salts, i.e. water table level, groundwater salinity and texture. In addition, we used existing databases (http://erdoterkep.mgszh.gov.hu) of tree plantations describing forest tree species and stand age.

In this paper, the results collected at 31 forested and 14 associated control stands (surveyed between 17 July and 17 October 2012) are reported.

Of the 14 study sites, 7 (forest and control) were equipped with groundwater monitoring sensors, and 4 of these with meteorological stations. The selection of tree species reflects their relative frequency in the tree plantations of the Great Hungarian Plain (Standovár 2012).

Meteorological stations were set up in four control stands (Mikepércs, Jászberény, Hajdúsámson and Nyírgyulaj), which recorded every 15 min air temperature, relative humidity, solar radiation and amount of rainfall.

Soil sampling was performed once and was scheduled for the late summer and early fall (September to October) when water table levels were the lowest. To avoid edge effects, boreholes for soil sampling and groundwater monitoring were located at least 50 m away from the border of plantations in both forested and control plots. The maximum depth of the boreholes was 11 m as limited by the augering equipment. Soil sampling was done to the depth of groundwater table + 1 m. ‘Soil’ as used in this paper includes the vadose zone. The samples were taken at every 20 cm increment from the topsoil (0–1 m), and at every 0.5 m increment <1 m.

Water samples were taken with a hand vacuum pump in cases when water table levels were <10 m and samples were stored at 4–5 °C until laboratory analysis. Field pH and EC measurements were performed on all groundwater samples. In forest stands, tree biomass was estimated based on tree height and stem circumference measurements (at a height of 130 cm). Tree biomass per unit area was calculated from these data for each tree species separately. Table 2 lists the methods used in this study.

**Table 2.** List of parameters and measurement methods used in this study. *Hygroscopicity refers to water content retained in the soil at 1.6 × 10^6^ cm tension with the method of Di Gléria *et al.* (1962, p. 301). The value is proportional with soil clayiness. **From these data we could estimate the amount of groundwater consumed and transpired by the given forest with the method of Gribovszki *et al.* (2008).

	Parameter	Measurement method	
		Field measurement	Laboratory measurement
**1**	***Climatic factors***		
A	Precipitation	Measurement with meterological stations (only at selected monitoring wells)	
B	Wind speed		
C	Relative humidity		
D	Solar radiation		
E	Air temperature		
**2**	***Hydrogeological factors***		
A	Geomorphology		
	Shallow or deep water table level	Water table level found during boring and stabilized after boring	
	Temporal water table level fluctuation (short term, long term)	Continuous water table level monitoring at seven sample sites—time series of water table level data	
B	Lithology		
	Soil water retention and soil permeability	Saturated soil conductivity estimation (only at monitoring wells)—calculation of effective porosity	Determination of soil particle size distribution with pipette (only at monitoring wells), hy1* (hygroscopicity)

C	Groundwater chemical composition	EC, pH (from all groundwater samples)	EC, pH, pNa, pCl (from all groundwater samples), concentrations of main anions and cations (only at monitoring wells)
**3**	***Biological factors***		
A	Evapotranspiration capacity: high, e.g. forest, low, e.g. grassland	Forest assessment (tree height measurement, trunk circumstance measurement (at the height of 130 cm)—biomass calculation (based on wood capacity curve, where tree species, tree height and trunk circumstance are known)**	
B	Salt tolerance: salt-tolerant or salt-sensitive tree		From publications
**4**	***Soil factors***		
A	EC	With pH and EC electrodes in 1 : 2.5 soil : water suspension	
B	pH		
C	pNa		With ion-selective electrode in the above suspension
D	pCl		
E	CaCO_3_		Scheibler calcimeter
F	Organic C		Turin method

Water table monitoring wells were augered and PVC tubes (8 cm diameter), extending 1 m below the water table level, were closely fitted inside each borehole. Water table levels measured with a pressure transducer (Model DA-LUB 222 by the manufacturer Dataqua) and meteorological data were recorded and stored with a datalogger (Model Hyga by the manufacturer EWS Bt) every 15 min. Water table levels of forest and control stands were compared after determining the ground elevation difference between the wells. The riparian-zone groundwater ET estimation technique described by Gribovszki *et al.* (2008) was used for ET calculation, based on the diurnal fluctuations of the groundwater levels (by further developing the original White (1932) method). As a comparative reference, Penman–Monteith ET (ET_PM) rates (for a grass reference surface) were calculated from the meteorological dataset (Allen *et al.* 1998).

Mean soil salinity (dS m^−1^) of forested and control stands by three depth increments (0–1 m, 1—water table depth and 0—water table depth) were analysed with ANOVA. Means and significant differences between group means are presented. Correlation coefficients between the three derived variables (difference between water table depth, soil salinity and groundwater salinity of forested and control stands) and biomass of the different tree species were calculated. Besides correlation coefficients, their significance levels were also indicated.

The relation between biomass and forest age with the differences in soil salinity between forested and control stands was analysed by linear regression. The statistical significance of the regression models is indicated with asterisks, as explained where relevant. The statistical analyses were performed by SPSS Version 17.

## Results

The comparison of tree plantations and grasslands/croplands showed a widespread water table depression and groundwater and soil salinization effect of tree planting (Table 3). The difference in average soil salinity between forested and control stands was significant (*P* < 0.05) at three depth increments according to one-way ANOVA, in which the differences between forest and control stands were compared separately for three depth increments (Table 4). The highest difference (0.053 dS m^−1^) was found between 1 m and the water table level where most of the tree roots were found. In the top metre an opposite trend was found with lower salinity in tree plantations (−0.041 dS m^−1^), yet not enough to compensate deep salinization, with full profiles showing a net increase (0.034 dS m^−1^).

**Table 3.** Number of cases when the hypotheses regarding differences between forested and control stands were proven correct (total number of cases measured for the given parameter is shown in brackets). Those cases are shown when the differences (EC of forest − EC of control) were positive.

Hypothesis on	Species			Total
	Poplar	Common oak	Black locust	
	12 cases	5 cases	14 cases	31 cases
Water table depth	9 (12)	4 (5)	10 (14)	23 (31)
Soil salinity	6 (11)	2 (5)	7 (11)	15 (27)
Water table salinity	10 (12)	4 (5)	11 (13)	25 (30)

**Table 4.** Mean soil salinity (dS m^−1^) of forested and control stands by depth increments with the results of ANOVA.

Depth	Stand		Significance
	Forest	Control	
0–1 m	0.106	0.147	0.008
1-m water table level	0.198	0.145	0.001
0-m water table level	0.176	0.142	0.016

Differences in average soil salinity values between forested and control stands along the full profile from the surface to the water table showed decreasing salinization for poplar, common oak and black locust (0.0484, 0.0304 and 0.0246 dS m^−1^, respectively), but were not significant. Moreover, the ratios of the water uptake (shown in Table 1 after Szodfridt 1993) to the differences in average soil salinity between forested and control stands were similar (14.050 m^2^ dS^−1^ for poplar, similarly 14.507 m^2^ dS^−1^ for common oak and 11.098 m^2^ dS^−1^ for black locust). These preliminary results suggest that water uptake rate is the most likely factor affecting the subsurface salt accumulation in the present situation.

**Figure 4.**
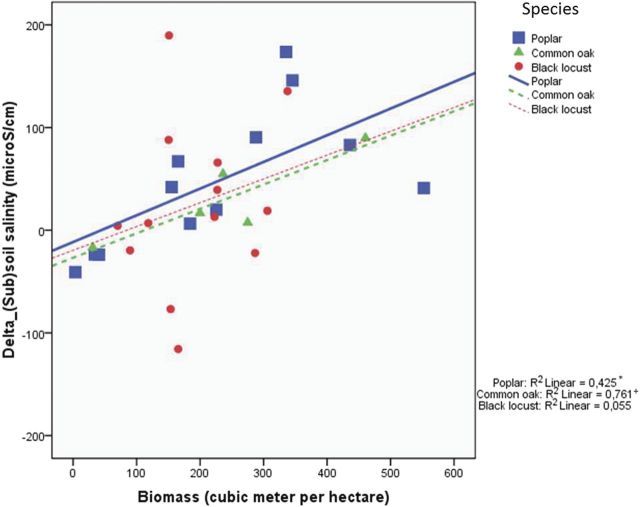
Scatter plot of tree biomass and the difference in average soil salinity between forested and control stands showing the adjusted lines for each species.

The highest correlation coefficients between the three derived variables in Table 3 and the biomass of the three studied tree species were found for the difference in average soil salinity between forested and control stands (Table 5). This variable is reported in more detail in Fig. 4, which illustrates the situation characterized by the correlation coefficient of 0.48 (Table 5), and presents the relation between biomass and differences in soil salinity between forested and control stands. The correlation coefficient increased in the same order as the water uptake (Table 1) from black locust through common oak to poplar. Furthermore, drawing the regression lines predicting differences of soil salinity between forested and control stands with biomass data (Fig. 4) shows that the slope of the regression lines increased in the order of poplar > common oak > black locust. Since this order is the same as that of the water uptake (Table 1), it suggests that water uptake, characteristic for the studied tree species, determines the effect of tree biomass on the differences of soil salinity between forested and control stands.

**Table 5.** Correlation coefficients between the three derived variables and biomass of the separate tree species. ^+^Correlation is significant at the 0.10 level (two-tailed). *Correlation is significant at the 0.05 level (two-tailed). **Correlation is significant at the 0.01 level (two-tailed).

Derived variable: difference between forested and control stands	Species			Total
	Poplar	Common oak	Black locust	
	12 cases	5 cases	14 cases	31 cases
Water table depth	0.173	0.334	−0.325	0.027
Soil salinity	0.652*	0.872^+^	0.235	0.480**
Water table salinity	0.033	0.013	0.849**	0.256

**Figure 5.**
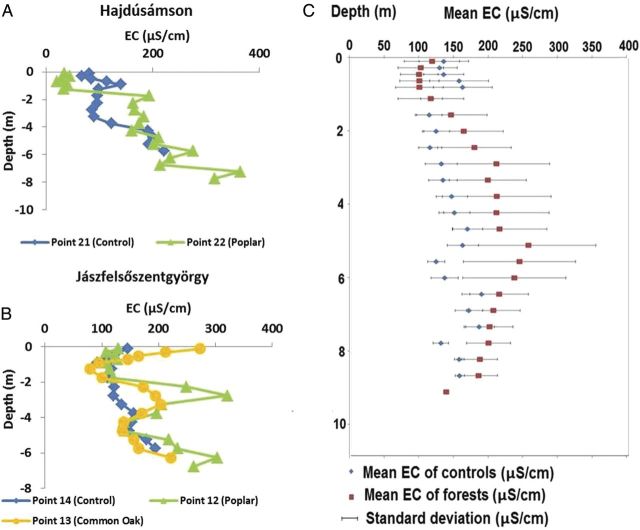
Typical depth profile of EC (μS cm^−1^) for forest and control stands at two monitoring sites (A and B), and (C) the depth profile of average EC values for forests and control plots (*n* = 31).

**Figure 6.**
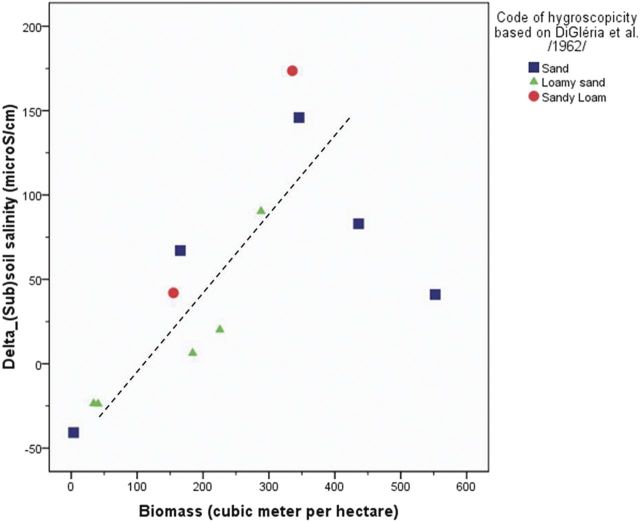
Scatter plot of tree biomass and the difference in average soil salinity between poplar and control stands. Labels indicate texture classes defined by hygroscopicity. Texture categories correspond to sand, loamy sand and sandy loam.

In order to detect the effect of other factors on the salt accumulation, the poplar stands were further analysed (see Fig. 5A and B as two examples). The linear relationship between the biomass and the difference in average soil salinity between poplar and control stands was close to linear (Fig. 6). There were outliers with sand texture. Indeed in sand, water movement is fast and both leaching and capillary rise go on quickly. On the other hand, the height of capillary rise is short.

**Figure 7.**
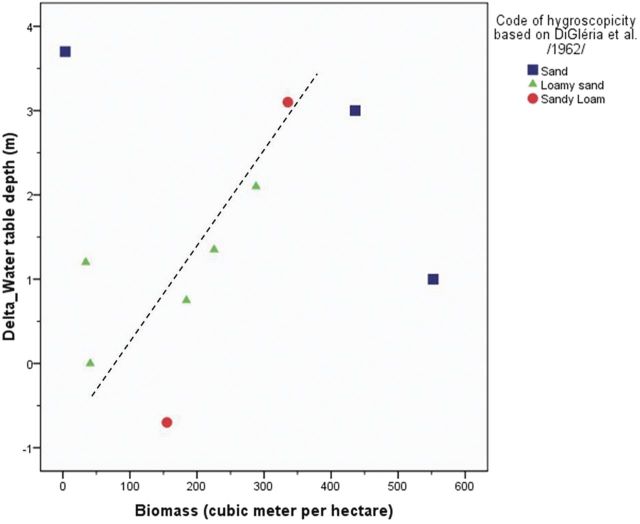
Scatter plot of tree biomass and the difference in water table depth between poplar and control stands. Texture categories correspond to sand, loamy sand and sandy loam.

This effect of sand texture was further investigated with the analysis of the scatter plot of tree biomass and the difference in water table depth between poplar and control stands (Fig. 7). Again the cases of sand texture were outliers, which support the previous observation that better linear relationship between salt accumulation/water table depression and biomass can be found on less coarse soils.

**Figure 8.**
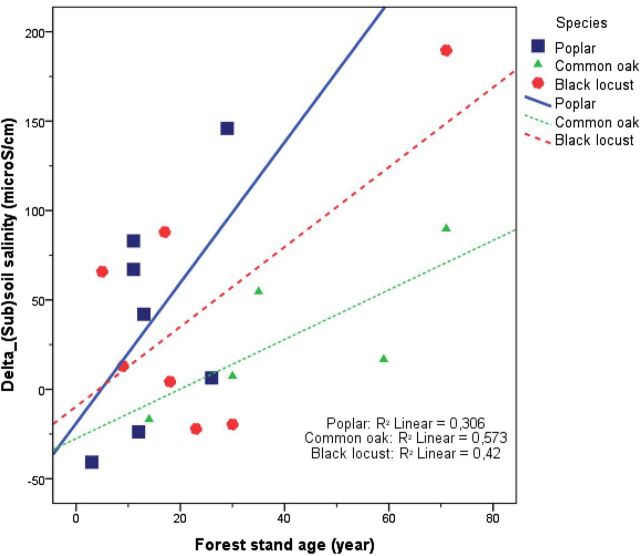
Scatter plot of forest stand age and the difference in average soil salinity between forested and control stands showing the adjusted lines for each species. The correlation coefficient calculated for the total data population was 0.466 (*P* = 0.044).

The forest stand age also showed a statistically significant relationship with the differences of soil salinity between forested and control stands (Fig. 8), which means that older forests accumulate more salt in the soil. The regression models of the three studied species were not significant at *P* < 0.05 level, but the slope of the equations shows an interesting order of poplar > black locust > oak, the same as the order of the vigour of growth (Table 1) as shown in Fig. 8. This figure suggests that vigour of growth determines the effect of forest stand age on the differences of soil salinity between forested and control stands.

**Figure 9.**
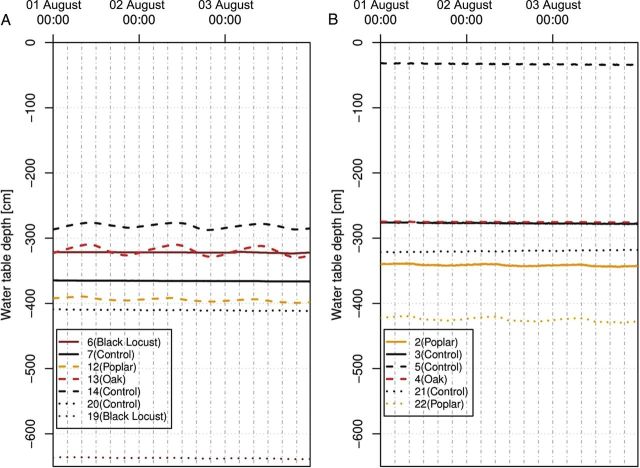
Water table level of monitoring wells on 1–3 August 2012. (A) Jászjákóhalma (6–7), Jászfelsőszentgyörgy (12–14), Nyírgyulaj-Máriapócs (19–20); (B) Nyírbogát (2–3), Debrecen-Mikepércs (4–5), Hajdúsámson (21–22).

In 74 % of the cases, the water table under the forest was deeper than under the control stand (Table 3). Daily fluctuations of water table levels were found at Jászfelsőszentgyörgy (Well No 13 under common oak, and Well No 12 under poplar), and Hajdúsámson (Well No 22 under poplar) (see Fig. 9). There were no fluctuations under black locust stands. The fact that daily fluctuations of water table levels were found under poplar and common oak supports the data on salt accumulation of soil as discussed above (Fig. 9A and B). The reason for the lack of daily water table level fluctuations under black locust stands might be 2-fold. Either the mostly young roots were too short to reach down to the water table or the water table was too deep. Mean water table depths were 5.53 [±2.22 SD] (poplar), 5.1 [3 SD] (common oak) and 6.53 [1.98 SD] (black locust). In fact, black locust is the tree species which was planted at the highest landscape positions compared with the other tree species.

**Figure 10.**
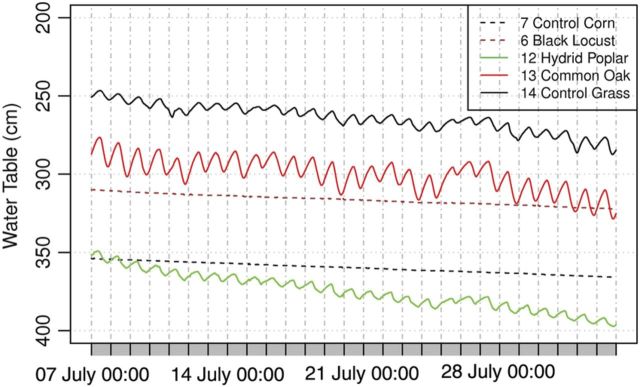
Changes in water table levels in Jászság between 7 July 2012 and 8 August 2012 (Well Nos 6 and 7 are from Jászjákóhalma, and Well Nos 12–14 are from Jászfelsőszentgyörgy).

In addition to daily fluctuations, there was a trend in water table changes, which were analysed in Jászság (Fig. 3) during the growth period between 7 July 2012 and 8 August 2012, a warm and rainless period. As shown in Fig. 10, there was 12- to 47-cm drop in water levels for each tree species. Whereas Well Nos 12–14 showed diurnal water table fluctuations, Well Nos 6 and 7 did not. The reason for the difference might be that Well Nos 12 and 13 were located under poplar and common oak stands, respectively. The root systems of these species characteristically consist of tap roots (Lucot and Bruckert 1992; Johansson and Hjelm 2012), which can reach down to the water table. However, the black locust (Well No 6, see Fig. 9A) has a tendency of growing roots laterally (Keresztesi 1968) and does not reach down to the 3-m deep water table. All chemistry data pointed to the effect of black locust on salt accumulation, which confirms that in the long term black locust from time to time utilized groundwater. In the grassland stand (Jászfelsőszentgyörgy Well No 14) there were water table fluctuations, but there were not in the maize field (Jászjákóhalma Well No 7), likely because of the differences in subsoil textures. In the fluctuation zone of the water table the texture classes were the following: Well No 6 Sand, No 7 Coarse Sand, No 12 Sandy Loam, No 13 Loam, No 14 Silty Loam, which would result in differences in the height of capillary rise (Di Gléria *et al.* 1962) among sites (Well No 14 > 13 > 12 > 6 > 7). In the stands of Jászfelsőszentgyörgy (Well No 12–14), the top border of capillary rise could reach the depth of 1–1.5 m promoting optimal water uptake of trees and consequently favouring water table drop compared with the control stand. In the stands of Jászjákóhalma, the highest capillary rise reached 3–3.5 m depth, which would not be high enough to provide water for the young black locust and maize, explaining the lack of water table fluctuations at these stands.

**Figure 11.**
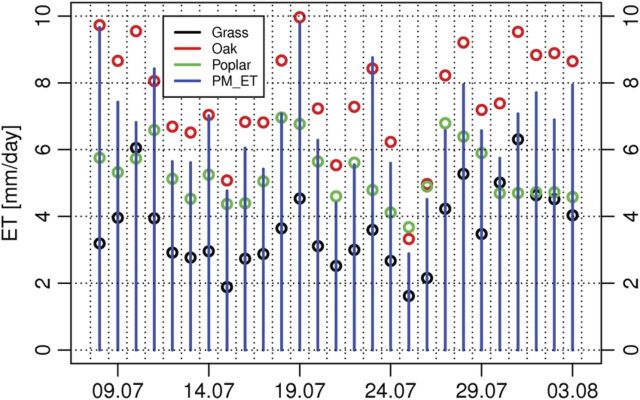
Daily groundwater ET (ET_gw_) rates compared with Penman–Monteith ET (PM_ET) values at Jászfelsőszentgyögy in July 2012.

The water use of different stands was compared using the example of the sampling sites of Jászfelsőszentgyörgy. Using an empirical method (Gribovszki *et al.* 2014), the groundwater uptake of different vegetation types in a very dry period of 2012 summer is presented in Fig. 11. The groundwater uptake of common oak site (mean 7.5 mm day^−1^) was slightly higher than reference ET_PM, (mean 6.6 mm day^−1^). The average groundwater ET (ET_gw_) of poplar stands (mean 4.7 mm day^−1^) was lower than common oak but higher than grass ET_gw_ (mean 3.5 mm day^−1^). It is important to highlight that the poplar stand was younger (13 vs. 59 years old) and had less tree biomass than the common oak stand (155 vs. 200 m^3^ ha^−1^). Oak ET rates were higher than estimates of reference potential ET calculated for a grass vegetation cover. This likely resulted because of the higher roughness of the forest canopy compared with grass, which would increase the aerodynamic conductance.

**Figure 12.**
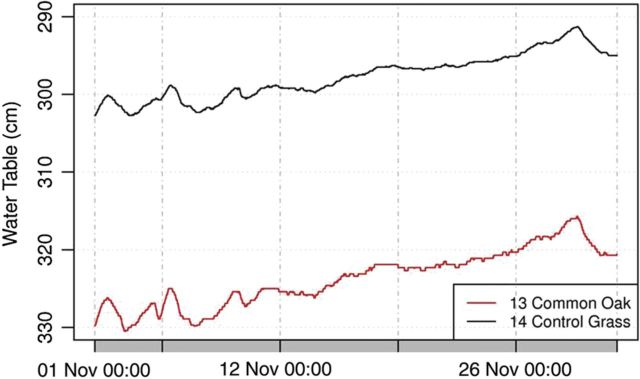
Water table level fluctuation at the start of the dormant period at the Jászfelsőszentgyörgy site (Well Nos 13 and 14) between 1 November 2012 and 2 December 2012.

Daily groundwater uptake seemed to be large, but taking into account that the period of analyses was very hot and moisture from the soil profile had been already lost (evaporated or taken up by roots) before July, the data seem to be acceptable. Because of the lack of transpiration, during the dormant vegetation period we would expect water table replenishment and water table level rise, which was observed at the start of the dormant period (Fig. 12). During the studied 1-month period (1 November 2012–2 December 2012) there was a rise of 13.5 (common oak) and 10.8 cm (control) in the water table levels. No daily water table fluctuations were observed during this period because of leafless canopy. During the dormant period, long-term water table changes could be observed, likely related to lateral groundwater flow and climatic effects. During this period, the water table level in the common oak stand was slightly higher (shallower) than in the control stand, with the differences of 4–7 cm.

## Discussion

Our basic hypotheses regarding differences between forested and control stands were proven correct for the slightly saline groundwater and coarse-textured soils of the Great Hungarian Plain. This experimental setup proved to be very useful for the study of biological factors (Fig. 2) of salinization. In our study, the water table was much less saline and the resulting salt accumulation was much lower than that found by Nosetto *et al.* (2007, 2008). Decreasing salinity in the top metre of tree plantations (Fig. 5C) suggested that the shallow root system of grasses and crops may contribute to strong local water uptake and accompanying salt accumulation, whereas the coarser and deeper roots of trees may favour leaching in the soil layer, as shown by Nosetto *et al.* (2007). The opposite pattern of salt accumulation under forest (topsoil low, subsoil high) and control plots (topsoil high, subsoil low) was also reported by He *et al.* (2014). Tree species had a specific effect on salinization, which was related to the water use (Fig. 4) and vigour of growth (Fig. 8) of the species. In these slightly salt-affected stands, the scheme of Fig. 2 should be changed. Instead of ‘salinity tolerance’, which was not an important factor in the present study, the vigour of growth should be listed. Such importance of forest productivity was emphasized by Lambert and Turner (2000, p. 119.), on the depth of water table.

Data collected on ET supported our hypothesis. For a comparison of ET values in Hortobágy (Hungary), Nosetto *et al.* (2007) determined groundwater ET rates of 1.9 mm day^−1^ (up to 3.2 mm day^−1^) for an oak forest on the basis of diurnal fluctuation of water table. In that experiment, the water table levels were significantly lower (on average 5 m) and the measurement period was in autumn therefore the higher values in our case were possible. Butler *et al.* (2007) determined groundwater ET (using diurnal method) rates of 2.9–9.3 mm day^−1^ for mixed vegetation-type based on continuous water table level readings, the water table depths were between 0.3 and 3.4 m from the surface, therefore calculated water table ET rates were close to total ET similar to our case.

The three species studied showed contrasting physiological properties, but even larger differences could be found when compared with the typical species planted in the Pampas, the eucalypt trees. Eucalypts are more (ca. twice) salt tolerant (Nosetto *et al.* 2008, Table 1) than common oak, the most salt-tolerant species in the Great Hungarian Plain. Compared with a case reported by Nosetto *et al.* (2008) in Argentina where evergreen eucalypt and pine forests provided almost continuous upward flux (with high vigour of growth, 1100–1500 mm year^−1^ water uptake), in Hungary, except for percolation of large rainfalls or flood water, there is regular leaching of the accumulated salts.

The proven relevance of the vigour of growth is a very important difference between the Hungarian and the Argentine situation reported by Nosetto *et al.* (2008). The reason for the replenishment of water table may be the total loss of forest canopy during the dormant period, facilitating the percolation of autumn winter and early spring precipitation through a very scarce understorey in planted forests. Since in grasslands there is complete cover of grasses or litter, it is not surprising that control stands had deeper water table levels during the dormant season. The reversal of the flow direction from upward in summer to downward in winter might have very strong consequence on salt accumulation of subsoil as also suggested by Lambert and Turner (2000).

## Conclusions

In areas where the hydrological balance is close to zero, any vegetation shift can result in large hydrological impacts. In the sub-humid Great Hungarian Plain, land use change from less (i.e. grassland, cropland) to more water-consuming vegetation type (i.e. forest) can result in water table drop and salt accumulation in both soil and groundwater.

Our findings agree with the overall guiding hypotheses regarding the hydrological effects of trees (Table 3), yet weaker support (56 % of total verified cases) was found for the soil salinization hypothesis. The general comparison of averaged soil salinity values with water table level proved our hypothesis that forest planting increases soil salinity but with a very small difference compared with those reported by Nosetto *et al.* (2007) (Fig. 4) and Nosetto *et al.* (2008) (Fig. 3). In deeper layers the larger water uptake by trees likely explained higher salt accumulation (Fig. 5).

We confirmed that forested sites on coarse-textured soil with slightly saline water table will be less susceptible to the hydrological effects of tree planting than found in the Pampas. Indeed, the magnitude of salt accumulation under the forest stands is far from being a threat to the trees because the original salt concentration of the groundwater was much lower than that found in clayey stands of Hungary and Argentina. In addition, the reversal of flow direction during the dormant period (i.e. downward water flux) would favour regular salt leaching and avoid strong salt accumulations in the Great Hungarian Plain.

Specific water uptake, characteristic for the three studied tree species, was assumed to determine the effect of tree biomass on the differences of soil salinity between forested and control stands. Tree biomass proved to be the best explanatory variable of salt accumulation. This suggests that the cumulative water use is a main driver of salt accumulation under trees, supporting the hypothesis of Lambert and Turner (2000). The faster the growth of trees, the stronger is the salinization process. In other words, similar levels of salinity will be achieved faster with fast growing species, and the link with biomass/water use underlies the key finding. This result points to a clear trade off between wood production (or carbon sequestration in biomass) and soil salinization.

Forest stand age also showed a statistically significant relationship with differences of soil salinity between forested and control stands. The mechanism behind the differences between tree species seems to be the differences in the vigour of growth. Tree morphology may also have an effect, because data suggested that taproots of poplar and common oak (Lucot and Bruckert 1992; Johansson and Hjelm 2012) facilitate groundwater uptake in these species. In contrast, the lateral and shallow roots of black locust (Keresztesi 1968) would not support groundwater uptake.

It was shown that the soil texture in the fluctuation depth zone of water table affects the water uptake of tree stands. Coarser texture classes (coarse sand and sand) will constrain net (downward–upward) groundwater use by vegetation and consequently there is (i) less marked effect on salt accumulation, (ii) less marked effect on water table depression and (iii) no water table fluctuations.

## Sources of Funding

Our work was supported by Országos Tudományos Kutatási Alapprogramok NN 79835 (Hungary) and EU COST FA0901.

## Contributions by the Authors

The first author devised the work plan, coordinated each element of the work, performed statistical analysis and wrote most of the text.

The second author worked in the field assessment, carried out most of the laboratory analysis and prepared the soil chemistry database. She prepared one preliminary version of the manuscript.

The third author worked in the field, collected the hydrological and meteorological data and prepared the hydrological database. He prepared figures and tables and contributed to writing the text.

The fourth author created the geographical information system for the selection of the sampling sites based on data on geology, groundwater chemistry, water table depth and texture classes.

The fifth author drew the overall scheme of the study. He developed the methodology of water table monitoring and soil chemistry study. He gave advice on the fieldwork and hydrological analysis. He reworked the first preliminary version of the manuscript.

The sixth author tested and refined the methodology of the chemistry and hydrology study. He helped to rework the first preliminary version of the manuscript.

The last author selected the monitoring stands. He developed the method for the determination of ET from groundwater, analysed and interpreted the hydrological data. He prepared figures and tables and contributed to writing the text.

## Conflicts of Interest Statement

None declared.
